# Winter weather controls net influx of atmospheric CO_2_ on the north-west European shelf

**DOI:** 10.1038/s41598-019-56363-5

**Published:** 2019-12-27

**Authors:** Vassilis Kitidis, Jamie D. Shutler, Ian Ashton, Mark Warren, Ian Brown, Helen Findlay, Sue E. Hartman, Richard Sanders, Matthew Humphreys, Caroline Kivimäe, Naomi Greenwood, Tom Hull, David Pearce, Triona McGrath, Brian M. Stewart, Pamela Walsham, Evin McGovern, Yann Bozec, Jean-Philippe Gac, Steven M. A. C. van Heuven, Mario Hoppema, Ute Schuster, Truls Johannessen, Abdirahman Omar, Siv K. Lauvset, Ingunn Skjelvan, Are Olsen, Tobias Steinhoff, Arne Körtzinger, Meike Becker, Nathalie Lefevre, Denis Diverrès, Thanos Gkritzalis, André Cattrijsse, Wilhelm Petersen, Yoana G. Voynova, Bertrand Chapron, Antoine Grouazel, Peter E. Land, Jonathan Sharples, Philip D. Nightingale

**Affiliations:** 1 Plymouth Marine Laboratory, Plymouth, UK; 2 University of Exeter, College of Life and Environmental Sciences, Exeter, UK; 3 National Oceanography Centre, Southampton, UK; 4 Ocean and Earth Science, University of Southampton, Southampton, UK; 5 Centre for Environment Fisheries and Aquaculture Science (Cefas), Lowestoft, UK; 6 National University of Ireland, Galway, Ireland; 7 Agri-Food and Biosciences Institute, Belfast, UK; 8 Marine Scotland Science (MSS), Aberdeen, UK; 9 The Marine Institute, Galway, Ireland; 10 Station Biologique de Roscoff, UMR CNRS - UPMC 7144 - Equipe Chimie Marine, Roscoff, France; 11 University of Groningen, Faculty of Science and Engineering, Groningen, Netherlands; 12 Alfred Wegener Institute, Helmholtz Centre for Polar and Marine Research, Bremerhaven, Germany; 13 Geophysical Institute, University of Bergen and Bjerknes Center for Climate Research, Bergen, Norway; 14 NORCE Norwegian Research Centre, Bjerknes Center for Climate Research, Bergen, Norway; 15 GEOMAR Helmholtz Centre for Ocean Research Kiel, Kiel, Germany; 16 Sorbonne Universités (UPMC, Univ Paris 06)-IRD-CNRS-MNHN, LOCEAN, Paris, France; 17 Institut de Recherche pour le Développement (IRD), centre de Bretagne, Plouzané, France; 18 VLIZ Flanders Marine Institute, Ostend, Belgium; 19 Helmholtz Zentrum Geesthacht, Centre for Materials and Coastal Research, Geesthacht, Germany; 20 Institut Francais Recherche Pour ĹExploitation de la Mer, Pointe du Diable, 29280 Plouzané, France; 21 University of Liverpool, School of Environmental Sciences, Liverpool, UK; 22 Present Address: School of Environmental Sciences, University of East Anglia, Norwich, UK; 23 Present Address: Geophysical Institute, University of Bergen and Bjerknes Center for Climate Research, Bergen, Norway

**Keywords:** Carbon cycle, Marine chemistry

## Abstract

Shelf seas play an important role in the global carbon cycle, absorbing atmospheric carbon dioxide (CO_2_) and exporting carbon (C) to the open ocean and sediments. The magnitude of these processes is poorly constrained, because observations are typically interpolated over multiple years. Here, we used 298500 observations of CO_2_ fugacity (fCO_2_) from a single year (2015), to estimate the net influx of atmospheric CO_2_ as 26.2 ± 4.7 Tg C yr^−1^ over the open NW European shelf. CO_2_ influx from the atmosphere was dominated by influx during winter as a consequence of high winds, despite a smaller, thermally-driven, air-sea fCO_2_ gradient compared to the larger, biologically-driven summer gradient. In order to understand this climate regulation service, we constructed a carbon-budget supplemented by data from the literature, where the NW European shelf is treated as a box with carbon entering and leaving the box. This budget showed that net C-burial was a small sink of 1.3 ± 3.1 Tg C yr^−1^, while CO_2_ efflux from estuaries to the atmosphere, removed the majority of river C-inputs. In contrast, the input from the Baltic Sea likely contributes to net export via the continental shelf pump and advection (34.4 ± 6.0 Tg C yr^−1^).

## Introduction

Temperate continental shelf seas (<200 m depth) occupy ~7% of the ocean surface area, yet contribute disproportionately to net marine drawdown of atmospheric carbon dioxide (CO_2_), accounting for 10–20% of the global total net uptake^1–3^. Century-scale storage of this CO_2_ in sediments and seawater results in a substantial climate regulation service, but has also caused ocean acidification (OA), a reduction in pH which is projected to continue as atmospheric CO_2_ increases^4^. Net atmospheric CO_2_ drawdown in temperate shelf seas is mediated by physical processes (dissolution of CO_2_) and nutrient-limited net community production (NCP: photosynthesis minus mineralization). Predictions of the future drawdown of atmospheric CO_2_ are highly contradictory. On the one hand, increasing CO_2_ in the atmosphere is expected to enhance the air to sea flux of CO_2_, simply by driving the respective concentration gradient. However, the inverse dependence of CO_2_ solubility on temperature^5^ may limit this flux as sea surface temperature (SST) increases. In contrast with observations showing a 35-year increase in shelf sea CO_2_ drawdown over the NW European shelf^6^, models predict a future decrease as a result of reduced nutrient supply and net biological CO_2_ uptake^7,8^. Other models suggest that saturation of the ‘continental shelf pump’ [CSP; the seasonal C export into intermediate waters of the open ocean^9^] may limit further drawdown from the atmosphere^10^.

Net air to sea CO_2_ flux may be calculated from ship-board observations of CO_2_ fugacity (fCO_2_; the partial pressure of CO_2_ corrected for its non-ideal-gas behaviour). Here, we evaluate the air to sea CO_2_ flux for the NW European shelf based on the most geographically-extensive, single-year, fCO_2_ dataset to date. Compared to previous evaluations (based on regional data extrapolation and combination of multiannual observations), this unique dataset provides a detailed snapshot of the air to sea CO_2_ flux.

The NW European shelf is hydrologically linked to the surrounding land-mass where temperature, precipitation and river-runoff are projected to increase over the next 50 years^11,12^. Policies regulating the application of fertilisers and wastewater treatment^13–16^ have reduced the nutrient loading on land and river-borne inputs to the shelf and may thereby alter the biological component of the air to sea CO_2_ flux. Similarly, changes in riverine dissolved organic C (DOC) inputs have the potential to modify shelf C-budgets as DOC is mineralized in shelf seas and returned to the atmosphere as CO_2_^17^. The recent trend of decreasing DOC input from major UK rivers (since 2000) has been attributed to implementation of the urban sewage treatment directive^18^. This followed an earlier trend of increasing DOC flux from rivers across the northern hemisphere which was attributed to soil recovery from acid rain after implementation of sulphur emission policies^17^. A comprehensive, observation-based analysis of the relative magnitude of C-fluxes on the NW European shelf which would allow us to constrain the potential impact of these feedbacks is currently missing. In order to address this, we have constructed a balanced budget based on observed C-fluxes on the NW European shelf (including air to sea CO_2_ flux, riverine and Baltic Sea inputs, inorganic C accumulation, C-burial and export via the CSP and advection). This enables an investigation of the factors controlling the air to sea flux, in turn providing guidance on the impact of land management practices.

## Results

### Air to sea flux in 2015

**Figure 1:**
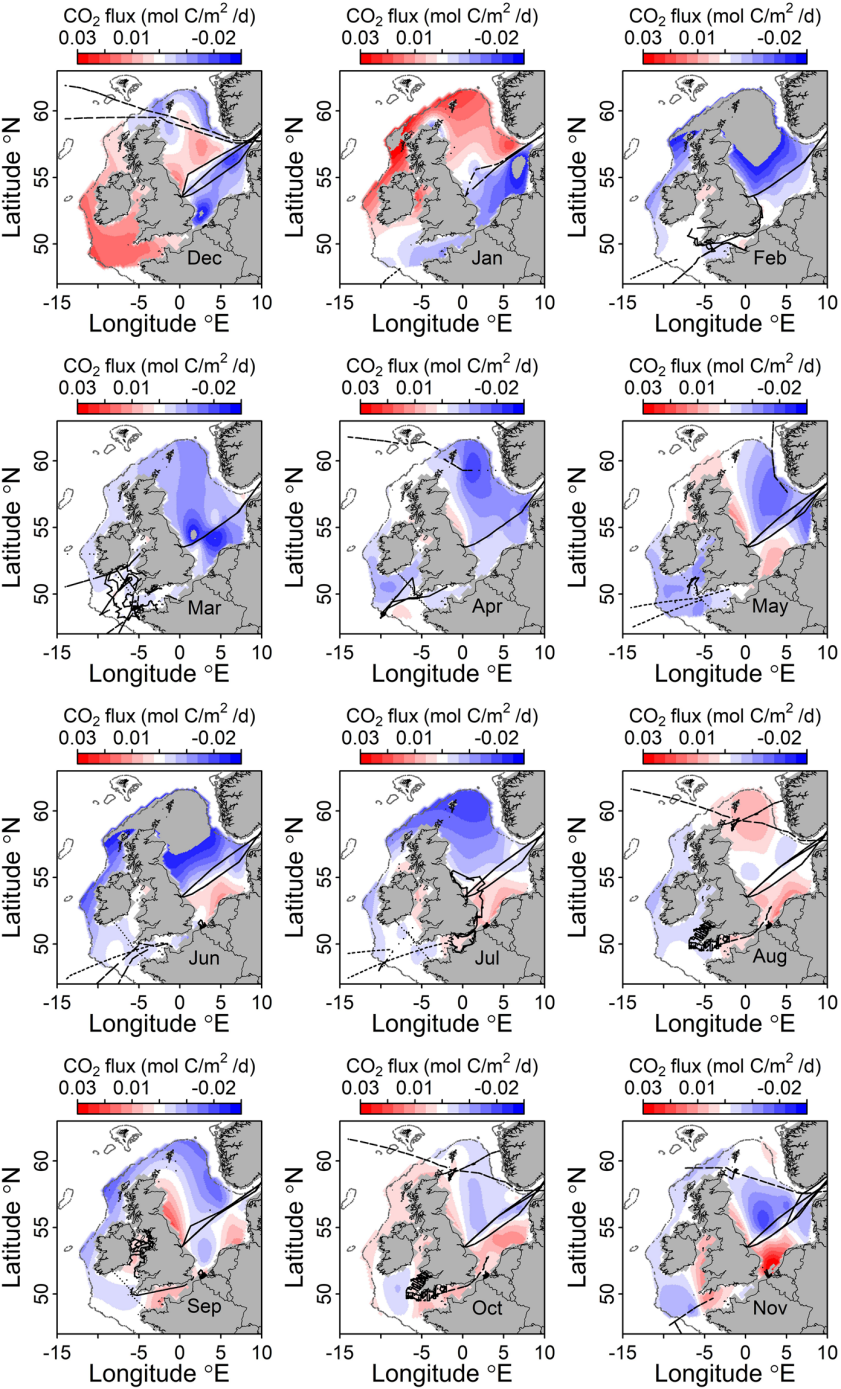
Monthly mean air-sea CO_2_ flux for the NW European shelf in 2015 (negative values denote influx from the air). Note that December is grouped with January-February in the first row (winter). Black dotted lines indicate ship tracks.

Monthly average fluxes showed considerable spatial and seasonal variability over 2015 (Fig. 1), reflecting changes in phytoplankton abundance, CO_2_ solubility and wind speed which generates turbulence at the air-sea interface promoting flux (Fig. 1 and Supplementary Figs. 1–3). The interplay between physical and biological processes can be used to explain the seasonal distribution of air to sea CO_2_ flux. For example, NCP contributes to the uptake of CO_2_. Variability in Chlorophyll-a^19^ was used as a proxy for NCP (Supplementary Fig. 1). Chlorophyll-a is weakly correlated with phytoplankton biomass so that monthly changes in the former are at least qualitatively related to NCP and by extension biological CO_2_ uptake or release^20^. A monthly increase in Chlorophyll-a thereby indicates net primary production (positive NCP), while a decrease suggests net respiration (negative NCP). In the Celtic Sea and central North Sea, high influx of CO_2_ from the atmosphere was found in spring/summer coincident with the highest surface Chlorophyll-a concentrations (correlation at −7.5 °E, 50 °N and 6 °E, 55 °N; Apr-Jul; R^2^ = 0.47; *p* < 0.05, *n* = 8). It is important to note that the distributions of air-sea CO_2_ flux and Chlorophyll-a are not perfectly anti-correlated, even during this net phytoplankton growth period, likely due to subsurface primary production in summer^21^, hysteresis in fCO_2 sea_ re-equilibration with fCO_2 air_^22^ and other factors. The apparently high levels of Chlorophyll-a found in winter (Dec. to Feb.), particularly in shallow coastal areas, are likely due to coloured organic matter and sediment reflectance rather than Chlorophyll-a [i.e., Case II waters^23^], (Supplementary Fig. 1). The solubility of CO_2_ (α_sea_ in Eq. 1; a function of seawater temperature and salinity; Supplementary Fig. 2) contributes to the potential in-water-CO_2_-concentration (fCO_2 sea_ in Eq. 1) and thus the ΔfCO_2_ (fCO_2 sea_ - fCO_2 air_). Thereby, lower α_sea_ in the southern North Sea in summer as well as remineralization of river-borne organic matter^24,25^ likely contribute to the efflux of CO_2_ to the atmosphere in this region. Finally, wind speed (U10; Supplementary Fig. 3), determines the gas transfer coefficient (*k* in Eq. 1). Wind speed was 49% higher in winter to early spring (Dec-Mar) compared to summer (Jun-Aug), resulting in 149% greater *k* values in winter and high drawdown of atmospheric CO_2_.

**Figure 2:**
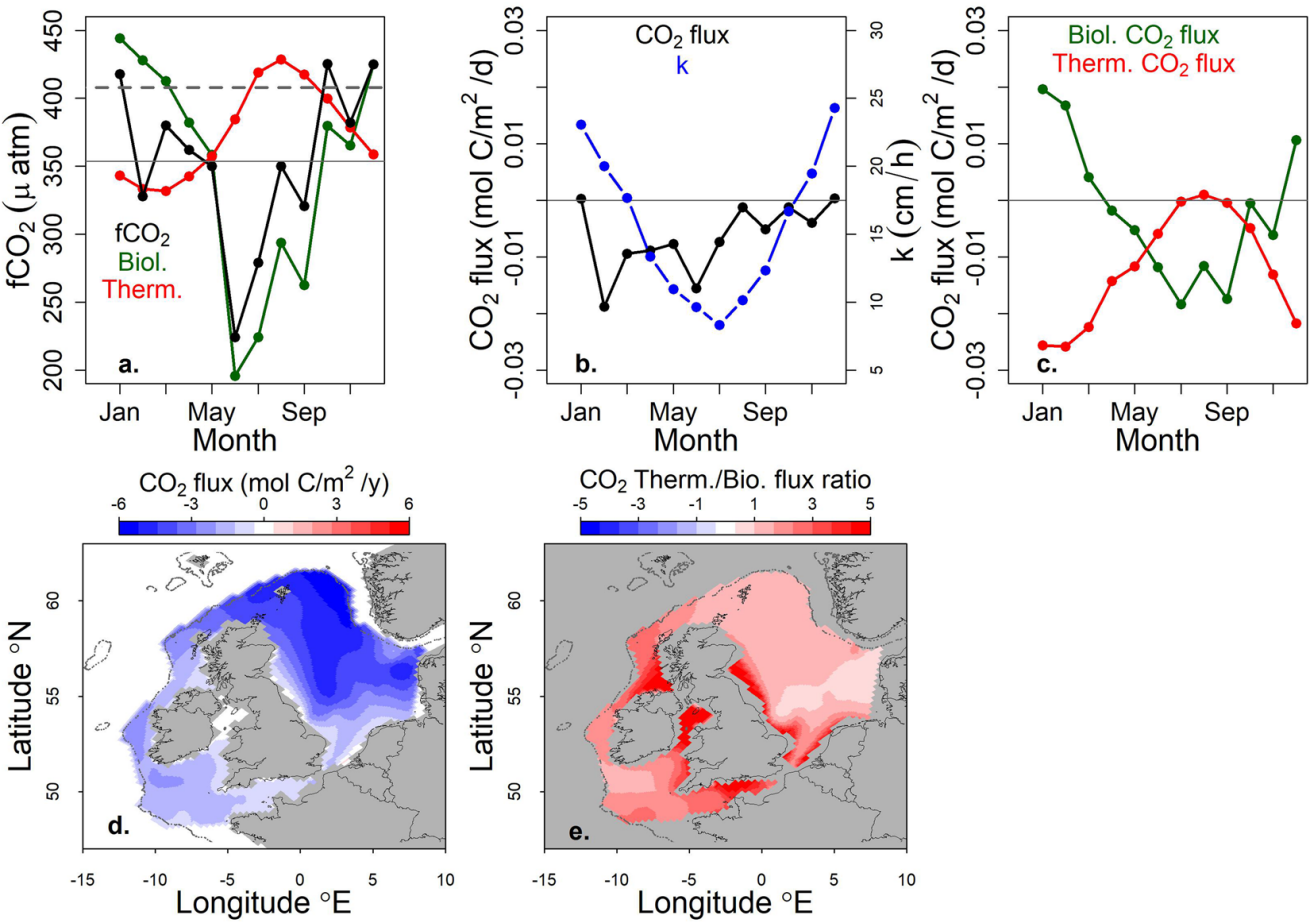
(**a**) Seasonal evolution of the mean fCO_2 sea_ (black) and its thermal (red) and biological + mixing (green) components (the solid and dashed horizontal lines represent mean seawater and atmospheric fCO_2_ respectively), (**b**) seasonal evolution of the mean air to sea CO_2_ flux (black) and the gas transfer velocity k (blue) (the horizontal line represents zero flux), (**c**) the thermal (red) and biological + mixing (green) components of the flux (the horizontal line represents zero flux), (**d**) annual air to sea CO_2_ flux (negative values denote influx from the atmosphere), (**e**) annual ratio of the thermal to biological + mixing components of CO_2_ flux (in our study, the ratio was only negative where the biological component was negative, i.e. the thermal component was always positive denoting influx).

The net-integrated air to sea flux of CO_2_ for 2015 within the region defined by the 200 m isobath was 26.2 ± 4.7 Tg y^−1^ (Fig. 2c). Including the Norwegian trench, the net influx of CO_2_ from the atmosphere was 26.7 ± 4.8 Tg y^−1^. fCO_2 sea_ and air to sea flux were further separated into thermal (e.g. due to cooling-enhanced solubility) and biological + mixing components following Eqs. 2 and 3. In 2015, the mean biological + mixing component (fCO_2 bio_) closely matched the seasonal pattern of fCO_2 sea_ over the domain with lower fCO_2 sea_ in spring/summer (Fig. 2a). In contrast the air to sea CO_2_ flux and its thermal component, were highest in winter, (Fig. 2b,c). A concomitant increase in the gas transfer velocity was evident in winter (Fig. 2b). The annual mean thermal to biological + mixing components ratio was thereby 2.1 ± 1.7 (where the uncertainty given is one standard deviation). The highest values for this ratio, indicating thermal-flux-component dominance, were observed in the western Hebridean Shelf, English Channel and northern Celtic Sea (Fig. 2d). This was largely due to low biological + mixing flux-component rather than high thermal-flux component in these regions. A notable exception was the Irish Sea where the net biological + mixing component was negative.

### C-budget terms

Table 1 lists the C-budget terms considered in our study (mean ± standard deviation of corresponding estimates listed in Table 1). The mean multi-year air to sea flux term was calculated as 23.0 ± 4.3 Tg C y^−1^. Baltic Sea C-inputs contribute 12.6 ± 2.7 Tg C y^−1^ to the NW European shelf: 89% as DIC, the remainder as organic-C. Rivers, were a smaller source of C contributing 2.4 ± 0.3 Tg C y^−1^ of DOC. The sum of pre-estuarine inputs was 21.6 Tg C y^−1^ while estuarine CO_2_ efflux to the atmosphere was calculated as 24.4 ± 10.4 Tg C y^−1^.

**Table 1:** C-fluxes into and out of (negative) the NW European Shelf (values in bold represent the mean ± standard deviation of the estimates listed below each flux).

Flux type	Flux (Tg C y^−1^)	Reference
River	${\boldsymbol{\Sigma }}(\overline{{\bf{DOC}}})$ 2.4 ± 0.3	
DOC	2.6	post-estuary; scaled from^24^
	2.1	pre-estuary; scaled from^26^
POC	0.6	pre-estuary; scaled from^24^
DIC	2.0	post-estuary; scaled from^24^, accounted in air-sea flux
	18.9	pre-estuary; scaled from^26^, accounted in estuarine- (24.4 ± 10.4 Tg C y^−1^) and shelf- air to sea flux
Baltic Sea	${\boldsymbol{\Sigma }}(\overline{{\bf{DOC}}}+\overline{{\bf{DIC}}})\,$12.6 ± 2.7	
DOC	0.8	^71^
	1.9	^72^
	1.1	^26^
	1.9	^73^
DIC	9.7	^72^
	12.7	^26^
Air to Sea	23.0 ± 4.3	
	26.2	this study
	26.9	Scaled from^28^
	19.8	Scaled from^25^,
	17.2	^29^
	25.0	Scaled from^26^ – as recalculated by^30^
DIC accum.	−1.0 ± 0.5	
	−0.6	for ΔpH = −0.0013 units y^−−1^ ^69^
	−1.0	for ΔpH = −0.0022 units y^−1^ ^42^
	−1.5	for ΔpH = −0.0035 units y^−1^ ^43^
Sed. burial	−1.3 ± 3.1	
	−1.8	scaled from^26^
	−0.9 ± 2.5	this study
Export	−34.4 ± 6.0	this study – mass balance assuming that 50% of river-DOC is mineralized and accounted for in the air to sea flux

Some terms are listed here for information but accounted in other terms and therefore do not contribute to the mean flux (e.g. the river-DIC input is accounted in the estuarine and shelf air to sea flux and therefore not included in the river-flux). The terrestrial input only considers dissolved organic carbon (DOC), as dissolved inorganic carbon (DIC) is implicitly accounted for in the air to sea flux. The air to sea flux estimate by^25^ was recalculated using 1.4 mol C m^−2^ y^−1^ for the North Sea, 0 mol C m^−2^ y^−1^ for the English Channel, and 1.0 mol C m^−2^ y^−1^ for the Celtic Sea, Faroes, North Scottish shelf, West Scottish shelf, West Irish shelf, Irish Sea and North Channel – and subsequently corrected for the wind reanalysis by Meyer *et al*. (2018). Note that the annual accumulation in DIC (DIC accum.) is negative here – otherwise it would count against export. Export via the CSP and advection was calculated as the difference between inputs and C-burial + OA, assuming that 50% of the river-DOC input is mineralized on the shelf (and therefore accounted for in the air to sea flux).

Our C-burial calculations showed that N_ret_ accounted for 63%, 91% and 97% of N_dep_ in mud, mud-sand and sand sediments respectively (each with an uncertainty of ±22%). Assuming that the conditions in March were representative of the low-productivity (i.e. net respirations) half of the year (winter) and the average of the May and August were representative of the high productivity half (spring and summer), we calculated annual net C-burial as 0.569 mol C m^−2^ y^−1^, 0.055 mol C m^−2^ y^−1^ and 0.008 mol C m^−2^ y^−1^ for mud, mud-sand and sand respectively. The annual net C-burial over the NW European shelf was 1.3 ± 3.1 Tg C y^−1^. This was therefore a small, but highly uncertain, loss term for shelf-sea C. The DIC accumulation rate, related to OA, was further scaled for the shelf area (1.06 × 10^12^ m^2^) and mean depth (78.3 m) to yield 1.0 ± 0.5 Tg C y^−1^. We acknowledge that this will vary regionally depending on the buffering capacity of seawater and other factors.

Finally, net exchange of C between the shelf and the open ocean via the CSP and advection was calculated as −34.4 ± 6.0 Tg C y^−1^ from the sum of the input terms (from the atmosphere, rivers and the Baltic Sea) minus C-burial and DIC accumulation. The uncertainty of our export-estimate is the standard error of the uncertainties in the other terms. Our estimate of the CSP is consistent with a previous estimate of 32.8 Tg C y^−1^ ^26^, which was based on the distribution of DIC in the North Sea. A further study estimated a monthly export of 2.2 Tg C via the Norwegian trench in late summer^27^, which yields 27.2 Tg C y^−1^ if extrapolated over the whole year.

## Discussion

Over the last two decades, a number of studies have quantified the air to sea flux of CO_2_ over the NW European shelf, regions within it or the wider European shelf^25,28–30^. When scaled to their respective areas, these estimates (including our own) fall in the range of 1.3–2.1 mol C m^−2^ y^−1^ with a mean value of 1.8 ± 0.3 mol C m^−2^ y^−1^ (Table 1). This is three-fold higher than the average open ocean influx of 0.60 mol C m^−2^ y^−1^ ^20^, but lower than some high latitude seas (e.g. the Barents Sea: 4 mol C m^−2^ y^−1^ ^31^) and upwelling systems (e.g. the South African coastal region: 3.83 mol C m^−2^ y^−1^ ^32^). Our study provides the first such estimate based on observations collected in a single year. This offers unique advantages for understanding inter-annual variability related to climate and weather. For example, previous work has shown that the North Atlantic Oscillation (NAO; the dominant climate mode over the North Atlantic and a major influence on weather in NW Europe) exerts a strong influence on fCO_2 sea_ distribution in the North Sea^33^. In the absence of comparable, annual data this could not be explored further (for reference, the 2015 winter-NAO index was positive, associated with above average precipitation and temperatures over our domain). We found that the 2015 influx of atmospheric CO_2_ was dominated by the winter months (January to March and December) despite the small air-sea concentration gradient during this period. This conclusion is further supported by the flux component analysis (thermal vs. biological + mixing) which showed thermal influx dominance over a whole year. Nevertheless, we acknowledge that the flux component analysis creates an artificial situation whereby the expected thermal summer-efflux does not materialize because of net biological drawdown of fCO_2 sea_ at that time. Peak influx of CO_2_ in winter, and/or due to high wind speeds and solubility, has also been observed in other shelf-seas^34^.

The paucity of single-year studies leaves a number of open questions relating to the inter-annual persistence of the pattern observed in 2015 where the winter air to sea CO_2_ flux was the dominant component of the net flux. Numerical models may be used to address this, but these are not entirely consistent with observations. Modelling studies for the NW European shelf are within ±50% of observation-based estimates [e.g. 1.5–2.6 mol C m^−2^ y^−1^ for the North Sea^7,35^ compared to 1.8 ± 0.3 mol C m^−2^ y^−1^ for observations^25,28–30^]. Wider disagreement is found for long-term climate projections of wind speed (e.g. CMIP5) where different models predict increase or decrease in extreme wind events^36^. If wind speed is critical in determining the overall annual influx of CO_2_ from the atmosphere (as shown here), it follows that model projections of this influx are equally contradictory and therefore uncertain.

**Figure 3:**
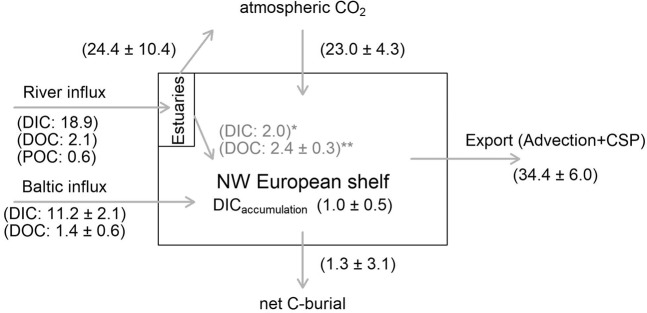
C-fluxes across the NW European shelf based on the present study and literature. Export is calculated as the difference between input/output terms and further subtracting the DIC accumulation term. (*): The post-estuarine DIC input is not considered in the calculation of export as it contributes to the air to sea flux and is therefore implicitly accounted for in this term. (**): We have arbitrarily assumed that 50% of post-estuarine river-DOC input is mineralized to CO_2_ on the shelf – this is implicitly accounted for in the air to sea flux and does not contribute to export.

Our synthesis of C-fluxes showed that the air to sea flux is the largest input term over the open NW European shelf (Fig. 3). River-borne C-inputs are largely lost in estuaries with CO_2_ efflux accounting for the majority of this loss. It should be noted that the estuarine CO_2_ efflux to the atmosphere is biased towards larger estuaries where measurements exist – these are also more urbanized/industrialised and have longer residence times for degassing and mineralization to take place. In smaller estuaries with short residence times the river-borne C-load may bypass the estuarine filter and contribute to the air-sea flux over the open shelf. Any river-DIC input which passes the estuarine filter (and therefore contributes to exchange with the atmosphere) will modify the shelf DIC and by extension shelf-fCO_2 sea_. Since this was measured through our observation network, the post estuarine DIC-input was implicitly accounted for in the air to sea flux over the shelf and should not be considered further in order to avoid double-counting. Mineralization of river-DOC to CO_2_ would likewise modify shelf-fCO_2 sea_ and be accounted for in the air to sea flux. The rapid decline of organic-C with distance offshore suggests that very little of this material contributes to export from the shelf^37,38^. The majority of organic-C from rivers must therefore be either buried or microbially/photochemically mineralized to CO_2_^24,26^. However, a recent study in the North Sea showed conservative mixing between DOM optical characteristics with increasing salinity suggesting simple dilution between river- and oceanic-DOM^37^. Some river-DOC must therefore be exported past the shelf and into the open ocean as indicated by the presence of terrestrial DOM in the deep ocean^39^. For the synthesis presented in Fig. 3, we therefore arbitrarily assume that half of post-estuarine DOC input is mineralized on the shelf (and accounted for in the influx from the atmosphere) while the other half contributes to export. Conversely, the Baltic input is likely to contribute more to the export and advection term, both as DIC and DOC because of the shorter residence time of this input in the North Sea. Most of the C exported from the NW European shelf exits through the Norwegian trench following the clockwise circulation around the British Isles, eastward flow through the English Channel and counter-clockwise circulation in the North Sea^26^. Consequently, the residence time in the Norwegian Trench (i.e. in proximity to the Baltic inflow) is in the order to 100 days compared to months/years elsewhere in the North Sea^40^. This difference in residence times allows for the Baltic C-input to be exported from the shelf via the CSP and advection to the north, while river-inputs are subject to outgassing and/or mineralization on the shelf.

Our calculation of net C-burial is consistent with previous work showing that most of the organic-C produced annually in shelf-seas is recycled rather than buried in sediments^41^. A previous estimate for North Sea net C-burial was within the uncertainty of our estimate [0.9 Tg C y^−1^ for the North Sea, scaled here for the larger surface area of our study = 1.8 Tg C y^−1^ ^26^]. Nevertheless, the C-burial term carries the largest proportional uncertainty. Although it is a relatively small term in this budget, net C-burial is of great interest as it provides a climate-regulation service by removing C from the contemporary C-cycle. The fate of C exported via the CSP and advection to the north is unclear, only providing a climate regulation service if it is entrained in intermediate and deep waters of the open ocean.

There is significant uncertainty in our estimate of the estuary to atmosphere CO_2_ flux. Conservation of mass dictates that if this term changed then export via the CSP and advection should also change (other input/output terms were better constrained in our analysis). Considering lower and upper limits for the estuarine efflux of CO_2_ to the atmosphere leads to the conclusion that the corresponding lower and upper limits for the export term would be 24 Tg C y^−1^ and 44.8 Tg C y^−1^ respectively. Independent estimates of the export term are consistent with our export estimate of 34.4 Tg C y^−1^. Our conclusion that nearly all of the supply of DIC to estuaries is lost to the atmosphere within estuaries, is therefore plausible. However there is considerable work to do on refining our estimates of this term. In this respect, it is important to note that our budget only considered estuarine and shelf ‘open water’ and did not include salt-marshes and other ‘blue carbon’ stores, which are important sinks for C, but beyond the scope of this study. The suggestion that the riverine input of DIC and DOC to estuaries is largely lost to the atmosphere within those systems means that the open shelf net air to sea CO_2_ flux is at present largely decoupled from terrestrial influence. Land-management policies which might alter the delivery of organic-C to the NW European shelf (e.g. restoration of peat-bogs) would only have a modest effect on the open-shelf air to sea CO_2_ flux, burial and export-terms since the river-DOC input is a relatively small term. Instead, the impact of varying river C-loads would likely be more pronounced in estuaries where some DOC-mineralization takes place and contributes to efflux of CO_2_. Changes in river nutrient concentrations driven by environmental policy would be expected to affect net primary production and concomitant drawdown of atmospheric CO_2_. However, if the majority of atmospheric CO_2_ influx is shown to consistently take place during winter (as shown here for 2015) then the impact of such policies (designed to reduce eutrophication and improve the health of the coastal environment) on the open shelf sink of atmospheric CO_2_ will be limited. As was the case for river-C loads, the impact of varying river nutrient loads is likely more pronounced in estuarine and near-shore environments.

Arguably the largest implications for the air to sea CO_2_ flux relate to global CO_2_ emission reduction policy. The most pertinent questions relate to the longevity of the shelf uptake of atmospheric CO_2_ when the latter is continuously increasing. Long-term studies have shown that the accumulation of DIC is more rapid on the NW European shelf than elsewhere, leading to higher rates of OA in the range of −0.0022 to −0.0035 pH units y^−1^ ^42,43^, compared to −0.0018 ± 0.0004 y^−1^ globally^44^. This may be evidence that export via the CSP and advection is becoming saturated in agreement with a modelling study which further predicted a negative feedback (decrease) on the air to sea flux^10^. Yet, comparing our 2015 air to sea CO_2_ flux to previous estimates based on observations in the 1990s and 2000s does not support a reduction in atmospheric CO_2_ influx. Continued international efforts under the global ocean acidification observing network (http://goa-on.org/), ICOS network^45^ and others are essential to help us understand, identify and ultimately predict changes in the annual shelf-sea uptake of atmospheric-CO_2_.

## Methods

**Figure 4:**
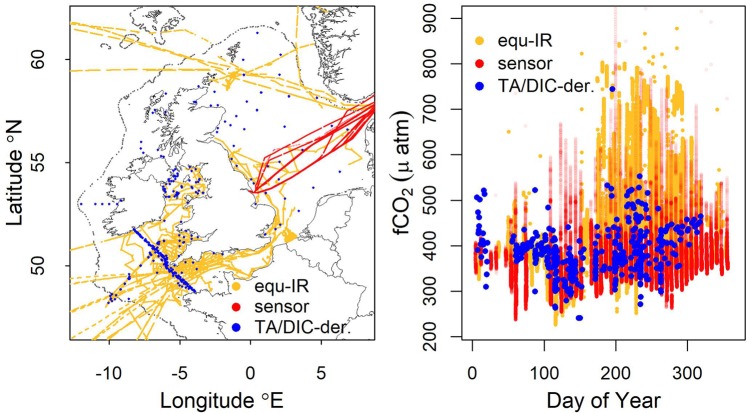
Spatial (left) and temporal (right) data coverage for fCO_2_ observations by three methods.

The NW European shelf is hereby defined as the region within the 200 m isobaths north of 47 °N and 8.5 °E. Based on a dataset of 298500 *in situ* observations of CO_2_ fugacity (Supplementary Table 1) we calculated the air to sea flux of CO_2_ using the open-source FluxEngine toolbox for 2015^46^. The dataset comprised three different method classes: a) continuous-flow equilibrator with partial drying of the headspace gas stream and infra-red detection following the recommendations of Pierrot *et al*.^47^ (equ-IR; 99.9 k observations), b) fCO_2_ derived from discrete measurements of Total Alkalinity and Dissolved Inorganic Carbon (TA/DIC-derived; 0.7 k observations) using the CO2SYS software package^48^ and c) fCO_2_ measured with a CONTROS HydroC CO_2_ flow-through sensor^49^ (sensor; 198 k observations) (Fig. 4). Further methodological details are given in the Supplementary Information.

Each method for the determination of fCO_2_ carries a certain analytical uncertainty defined by its respective precision and accuracy, which, in turn, are influenced by properties such as instrument-drift, calibration frequency, equilibration timescale and resolution^50,51^. In order to quantify the internal consistency of observations we examined ‘crossovers’ between different datasets and for the three method classes (see Supplementary Information for details). A crossover was defined as a maximum distance of 40 km between observations, using 30 km as the equivalent distance for one day (e.g. two data-points, one day apart in time and 10 km apart in space, yield a nominal equivalent ‘distance’ between data-points of 40 km). The crossover analysis revealed a correlation between the three different method classes (R^2^ > 0.998) with linear slopes (bias) statistically indistinguishable from unity. These statistics attest to the validity of the crossover analysis whilst the addition of ‘salinity and temperature agreement’ criteria (<0.2 and <0.5 °C respectively) further minimized the influence of river plumes and advection. There was therefore no systematic bias by any of the measurement techniques, which enabled us to use the whole dataset for calculating air-sea fluxes. We calculated a weighted uncertainty of 13.2 μatm for the whole dataset (note that this is not the accuracy which had a weighted mean value of 4 μatm based on the accuracy of individual systems; see Supplementary Information). Whilst we stress that this was not a strict inter-comparison of methods, it nevertheless provides confidence in the dataset consistency as a whole and provides a useful metric to propagate in the calculation of flux uncertainty.

All instantaneous and integrated air-sea gas fluxes were calculated using the freely available FluxEngine toolbox^46^. The air-sea flux of CO_2_ was calculated from the following equation:

1 $${\rm{F}}={\rm{k}}\times ({{\rm{\alpha }}}_{{\rm{sea}}}\times {{\rm{fCO}}}_{2{\rm{sea}}}-{{\rm{\alpha }}}_{{\rm{air}}}\times {{\rm{fCO}}}_{2{\rm{air}}}),$$

where k is the gas transfer velocity (units: cm h^−1^), α_sea_ is the solubility of CO_2_ at the bottom of the mass boundary layer and within the seawater (a function of salinity and temperature; units: g m^−3^ μatm^−1^), α_air_ is the equivalent solubility at the top of the mass boundary layer (the air-side solubility; units: g m^−3^ μatm^−1^), fCO_2 sea_ and fCO_2 air_ are the fugacity of CO_2_ in seawater and air respectively (units: μatm).

fCO_2 sea_ data were re-analyzed to be valid for a consistent sea surface (sub-skin) temperature dataset and depth^52^ using a climate quality (sub-skin) satellite temperature dataset^53^. These fCO_2 sea_ data (valid for the sub-skin and assumed to represent the bottom of the mass boundary layer) were then spatially interpolated (Data Interpolating Variation Analysis, DIVA v 4.7.1)^54^ to a 50 km polar stereographic grid. A cool skin correction of −0.17 °C was used^55^ to enable the calculation of skin temperature (using the sub-skin dataset as a reference) and thus the air-side solubility α_air_. Atmospheric fCO_2 air_ was calculated within FluxEngine using the air pressure at the sea surface (European Centre for Medium Range Weather Forecasting re-analysis; https://www.ecmwf.int/en/research/climate-reanalysis/era-interim) and *in-situ* CO_2_ dry air mole fraction from the National Oceanic and Atmospheric Administration Earth System Research Laboratory^56^. The atmospheric fCO_2_ dataset used here offers a well characterized, quality controlled, ‘clean’ and consistent dataset for the calculation of flux. Wind speed (U_10_) data were obtained from the Cross-Calibrated Multi-Platform (CCMP) version 2^57^. Salinity data came from the World Ocean Atlas 2013^58^. We used a quadratic wind driven parameterization of the gas transfer velocity^59^ that was originally developed using shelf sea measurements. All input data were re-gridded to the 50 km polar stereographic grid. Total uncertainties in the air to sea flux calculations solely due to the input data were calculated using an ensemble method. Twenty five sets of calculations were made, with fCO_2_, SST and U_10_ inputs perturbed by random noise representing the known uncertainties of the input parameters; σ(fCO_2_) = 13.2 μatm; σ(U_10_) = 0.44 ms^−1^; σ(SST skin and sub-skin) = 0.6 °C. This ensemble gave an air-sea flux uncertainty of ±2%.

In order to investigate uncertainties arising from the DIVA interpolation of fCO_2 sea_, we compared the interpolated outputs with independent fCO_2 sea_ data from a buoy in the Western English Channel (fCO_2 buoy_ at station L4 operated by PML and not included in the combined fCO_2_ dataset described above; see Supplementary Information for details). The corresponding daily air to sea flux (F_buoy_) was computed using the same parameters as in FluxEngine (i.e. with the same *k*, α_sea_, α_air_, and fCO_2 air_), apart from the fCO_2 sea_ field. The daily flux using fCO_2 buoy_ was not significantly different from the corresponding monthly flux using the combined fCO_2_ dataset described above (paired t-test; t = −9.34, *p* < 0.001, *n* = 184). Nevertheless, the 95% confidence interval 0.003 mol m^−2^ month^−1^ represented an uncertainty of 16% of the annual flux at L4 when extrapolated for the whole year. The maximum total uncertainty from mapping and the ensemble runs described above (perturbations of fCO_2_, U_10_ and SST) was therefore 18%. An additional 5% uncertainty arises from three quadratic parameterizations of *k* for the North Atlantic^60^. However, the ongoing debate regarding the optimum parameterization of *k* is beyond the scope of this paper and not considered further.

We separated the mean fCO_2_ for each 50 km grid cell into its biological + mixing (Eq. 2) and thermal components (Eq. 3)^20^.

2 $${{\rm{fCO}}}_{2{\rm{bio}}}={{\rm{fCO}}}_{2{\rm{sea}}}\times {{\rm{e}}}^{(0.0423\times (\overline{{\rm{SST}}}-{\rm{SST}}))},$$

3 $${{\rm{fCO}}}_{2{\rm{therm}}}=\overline{{{\rm{fCO}}}_{2{\rm{sea}}}}\times {{\rm{e}}}^{(0.0423\times ({\rm{SST}}-\overline{{\rm{SST}}}))},$$

where fCO_2_ and $\overline{{{\rm{fCO}}}_{2}}$ are the observed and annual mean fCO_2_ for each grid cell, SST and $\overline{{\rm{SST}}}$ are the observed and annual mean sea surface temperature respectively (sub skin). fCO_2 therm_ and fCO_2 bio_ were subsequently used to replace fCO_2 sea_ in Eq. 1 in order to derive the respective flux components.

C-burial in shelf sediments was calculated for three sites in the Celtic Sea: a) a muddy sediment (51.2114 °N, 6.1338 °W), b) a sandy sediment (51.0745 °N, 6.5837 °W) and c) a mud-sand sediment (station CCS: 49.4117 °N, 8.5985 °W). The following biogeochemical rates were determined at these sites in March (end of winter), May (during the spring bloom) and August 2015 (late summer): benthic oxygen consumption (Resp = respiration), nitrification (Nit), denitrification (Den), anammox (Ax) and sediment-water inorganic-N fluxes (FN; for NO_3_^−^ + NO_2_^−^ + NH_4_^+^) (e.g. Supplementary Table 3)^61^. Our general reasoning for the C-burial calculation relies on the conservation of mass and specified C:N ratios in organic matter, where near-closure of the C-cycle is assumed *a priori* and tested against closure of the N-cycle *a posteriori* (see Supplementary Information for calculations).

In order to provide context for the air to sea flux we further considered C-inputs to the NW European shelf from rivers and the Baltic inflow as well as loss-terms from the shelf (estuarine degassing, C-burial in sediments and export via the CSP and advection). For this purpose, we constructed a balanced budget where the CSP+ advection term were the remainder of all other terms.

Firstly, we combined our 2015 estimate of air to sea CO_2_ flux with previous studies to obtain a mean influx term from the atmosphere^25,28–30^.

River-borne C enters the NW European shelf in a buoyant, low-salinity layer and must first transit through estuaries where CO_2_-outgassing as well as organic-C burial and/or mineralization moderate the river-borne C-input to the shelf^62–65^. We therefore used a previous estimate of C-export from European rivers north of 50 °N (5.4 × 10^−6^ Tg C y^−1^ km^−2^; after the estuarine filter)^24^, scaled to the catchment area of rivers in our domain [892 × 10^3^ km^2^ ^66^] to estimate a post-estuarine input of 4.8 Tg C y^−1^_._ This was further separated into DIC (41.1%), DOC (54.3%) and POC (4.4%)^24^. The latter (0.2 Tg C y^−1^) is thought to be trapped in estuaries where it contributes to C-burial and outgassing^24^ and is therefore not considered further as an input to the NW European shelf. By comparison, the freshwater-endmember (i.e. pre-estuarine) of C-inputs to the North Sea^26^, scaled to the shelf area in our domain, yielded 18.9 Tg C y^−1^ and 2.1 Tg C y^−1^ for DIC and DOC respectively. The estimates of Ciais *et al*. (2008) and Thomas *et al*. (2005) are roughly in agreement for DOC, but differ by one order of magnitude for DIC. However, correcting the higher estimate of Thomas *et al*. (2005) for CO_2_ outgassing could easily account for this difference. We estimate the estuarine efflux of CO_2_ to the atmosphere as 24.4 ± 10.4 Tg C y^−1^, based on: a) estuarine sea to air efflux of 19.8–62.0 mol C m^−2^ y^−1^^25,62,65,67^; b) the ratio of estuarine area to coastline length of 2.64 km^2^ km^−1^ ^68^; and c) the coastline length of the domain considered here (18204 km).

As a result of long-term, net CO_2_ influx from the atmosphere and lateral advection from the open ocean, the NW European shelf is gaining C in the form of DIC which causes Ocean Acidification (OA). Whilst this is not an input term, it does not contribute to export via the CSP+ advection and must therefore be accounted for as the latter is the remainder of input vs output terms. In our study, this DIC accumulation rate was estimated from annual OA rates in the range of - 0.0013 to −0.0035 pH-units^42,43,69^. We estimated a mean DIC accumulation rate of 1.01 µmol kg^−1^ y^−1^ using the CO2SYS software^48^ with pH_T_ = 8.05 and Total Alkalinity of 2283.1 µmol kg^−1^ ^70^ as baseline.

## Supplementary information

Supplementary Information

## Data Availability

All fCO_2_ data used in this study are available from the SOCAT and Ferrybox databases (www.socat.info and www.ferrybox.org).
