# Supplementary material for: Winter weather controls net influx of atmospheric CO2 on the north-west European shelf
Source: Sci Rep. 2019 Dec 27;9:20153. doi: 10.1038/s41598-019-56363-5 (PMC6934492; doi:10.1038/s41598-019-56363-5)
Supplement: Supplementary file 1 — Supplementary Information [file 41598_2019_56363_MOESM1_ESM.docx]

Supplementary Information

Winter weather controls net influx of atmospheric CO_2_ on the north-west European shelf.

Vassilis Kitidis ^1^, Jamie D. Shutler ^2^, Ian Ashton ^2^, Mark Warren ^1^, Ian Brown ^1^, Helen Findlay ^1^, Sue E. Hartman ^3^, Richard Sanders ^3^, Matthew Humphreys ^4, †^, Caroline Kivimäe ^3^, Naomi Greenwood ^5^, Tom Hull ^5^, David Pearce ^5^, Triona McGrath ^6^, Brian M. Stewart ^7^, Pamela Walsham ^8^, Evin McGovern ^9^, Yann Bozec ^10^, Jean-Philippe Gac, ^10^, Steven M.A.C. van Heuven ^11^, Mario Hoppema ^12^, Ute Schuster ^2^, Truls Johannessen ^13^, Abdirahman Omar ^14^, Siv K. Lauvset ^14,^ ††, Ingunn. Skjelvan ^14^, Are Olsen ^13^, Tobias Steinhoff ^15^, Arne Körtzinger ^15^, Meike Becker ^15,^ ††, Nathalie Lefevre ^16^, Denis Diverrès, ^17^, Thanos Gkritzalis ^18^, André. Cattrijsse ^18^, Wilhelm Petersen ^19^, Yoana Voynova ^19^, Bertrand Chapron, ^20^, Antoine Grouazel, ^20^, Peter E. Land, ^1^, Jonathan Sharples ^21^, Philip D. Nightingale ^1^

^1^ Plymouth Marine Laboratory, Plymouth, UK

^2^ University of Exeter, College of Life and Environmental Sciences, Exeter, UK

^3^ National Oceanography Centre, Southampton, UK

^4^ Ocean and Earth Science, University of Southampton, Southampton, UK

^5^ Centre for Environment Fisheries and Aquaculture Science (Cefas), Lowestoft, UK

^6^ National University of Ireland, Galway, Ireland

^7^ Agri-Food and Biosciences Institute, Belfast, UK

^8^ Marine Scotland Science (MSS), Aberdeen, UK.

^9^ The Marine Institute, Galway, Ireland

^10^ Station Biologique de Roscoff, UMR CNRS - UPMC 7144 - Equipe Chimie Marine, Roscoff, France

^11^ University of Groningen, Faculty of Science and Engineering, Groningen, Netherlands

^12^ Alfred Wegener Institute, Helmholtz Centre for Polar and Marine Research, Bremerhaven, Germany

13 Geophysical Institute, University of Bergen and Bjerknes Center for Climate Research, Bergen, Norway

^14^ NORCE Norwegian Research Centre, Bjerknes Center for Climate Research, Bergen, Norway

^15^ GEOMAR Helmholtz Centre for Ocean Research Kiel, Kiel, Germany

^16^ Sorbonne Universités (UPMC, Univ Paris 06)-IRD-CNRS-MNHN, LOCEAN, Paris, France

^17^ Institut de Recherche pour le Développement (IRD), centre de Bretagne, Plouzané, France

^18^ VLIZ Flanders Marine Institute, Ostend, Belgium

^19^ Helmholtz Zentrum Geesthacht, Centre for Materials and Coastal Research, Geesthacht, Germany

^20^ Institut Francais Recherche Pour ĹExploitation de la Mer, Pointe du Diable, 29280 Plouzané France

^21^ University of Liverpool, School of Environmental Sciences, UK

† School of Environmental Sciences, University of East Anglia, Norwich, UK

†† Geophysical Institute, University of Bergen and Bjerknes Center for Climate Research, Bergen, Norway

Corresponding author: Vassilis Kitidis ([vak@pml.ac.uk)](mailto:vak@pml.ac.uk))

**Methods**

**fCO_2_ observations**

The combined 2015 fCO_2_ dataset comprised three different method classes: a) continuous-flow equilibrator with partial drying of the headspace gas stream and infra-red detection (equ-IR; 99.9 k observations), b) fCO_2_ derived from discrete measurements of Total Alkalinity and Dissolved Inorganic Carbon (TA/DIC-derived; 0.7 k observations) and c) fCO_2_ measured with a CONTROS HydroC CO_2_ flow-through sensor (sensor; 198 k observations) (Supplementary Table 1).

Supplementary Table 1: Data contributing organizations, research vessels and respective method class. Organisations are: AFBI (Agri-Food and Biosciences Institute); AWI (Alfred-Wegener-Institut); CEFAS (Centre for Ecosystems Fisheries and Aquaculture Science); GEOMAR (Helmholtz-Zentrum für Ozeanforschung Kiel); HZG (Helmholtz-Zentrum Geesthacht); LOCEAN (IRD-LOCEAN); MI (Marine Institute – Ireland); MSS (Marine Scotland Science); NOCS (National Oceanography Centre Southampton); PML (Plymouth Marine Laboratory); SBR (Station biologique de Roscoff); UoB (University of Bergen); UExeter (University of Exeter); VLIZ (Flanders Marine Institute).

| Research Organisation | Vessel | Method | Reference |
| --- | --- | --- | --- |
| AFBI | RV Corystes | TA/DIC-derived | ^1^ |
| AWI | RV Polarstern | equ-IR | ^2^ |
| CEFAS | RV Endeavour | equ-IR | ^3^ |
| CEFAS | RV Endeavour | TA/DIC-derived | ^1^ |
| GEOMAR | Atlantic Companion | equ-IR | ^4^ |
| HZG | Lysbris Seaways | sensor | ^5^ |
| LOCEAN | Cap San Lorenzo | equ-IR | ^6^ |
|  | Colibri | equ-IR | ^6^ |
| MI | RV Celtic Explorer | TA/DIC-derived | ^1^ |
|  | RV Celtic Voyager | TA/DIC-derived | ^1^ |
| MSS | RV Scotia | TA/DIC-derived | ^1^ |
| NOCS |  | TA/DIC-derived | ^1^ |
| PML | RRS Discovery | equ-IR | ^3^ |
|  | RV Plymouth Quest  RV Plymouth Quest | equ-IR  TA/DIC-derived | ^7^ |
| SBR | Pont Aven | TA/DIC-derived | ^8^ |
| UoB | GO Sars | equ-IR | ^9^ |
|  | Nuka Arctica | equ-IR | ^9^ |
| UExeter | Benguela Stream | equ-IR | ^10^ |
| VLIZ | Simon Stevin | equ-IR | ^11^ |

The equ-IR data were collected using standard techniques following the recommendations of Pierrot et al. ^4^. The reader is referred to the references in Supplementary Table 1 for further detail regarding individual instruments. Briefly, the instrumentation comprised different designs of a continuous flow equilibrator (1-8 L; with a flow rate of 1-3 L min^-1^), partial drying of the headspace gas-stream (Peltier and/or Nafion driers) and non-dispersive infrared spectrometer (LiCOR; models LI-6262; LI-840; LI-7000). The instruments were calibrated against gas standards traceable to the World Meteorological Organization. The equ-IR method class has a nominal accuracy of 2 μatm, although this increases to 4 μatm in coastal waters where sharp gradients in salinity and sea surface temperature persist (e.g. ^3^).

TA/DIC-derived fCO_2_ was calculated using the CO2SYS software package ^12^ with the H_2_SO_4_ dissociation constants of Dickson et al. (1990) ^13^ and the carbonic acid dissociation constants of Mehrbach et al. (1973) refitted by Dickson and Millero (1987) ^14,15^. TA/DIC sampling and analysis is described in detail by Hartman et al. ^1^. DIC and TA were measured coulometrically and by open cell potentiometric titration with HCl respectively, using standard methods ^16^. Samples and Reference Materials from A.G. Dickson (Scripps Institution of Oceanography) were analysed on a VINDTA 3C (Marianda, Germany) and Apollo SciTech (USA) instruments (DIC Analyzer AS-C3 and TA Titrator AS-ALK2). Precision and accuracy for replicate analyses was better than ±4.0 μmol kg^-1^ and ±3.9 μmol kg^-1^ for DIC and TA respectively. These translate to a combined analytical accuracy of 10 μatm fCO_2_ ^17^

The Contros Hydro-C sensor was integrated with an FSI FerryBox system on a containership crossing between Moss (Norway) and Immingham (UK) ^5^. Five different sensors were used in 2015. All sensors were corrected for ‘zero-drift’ and calibrated according to the manufacturer’s instructions. A laboratory inter-comparison of the Contros Hydro C sensor and a reference equ-IR system found good agreement over a wide range of conditions with an accuracy of 3.7 μatm pCO_2_ ^18^.

**Ancillary Figures: Chlorophyll-a, solubility and wind speed**


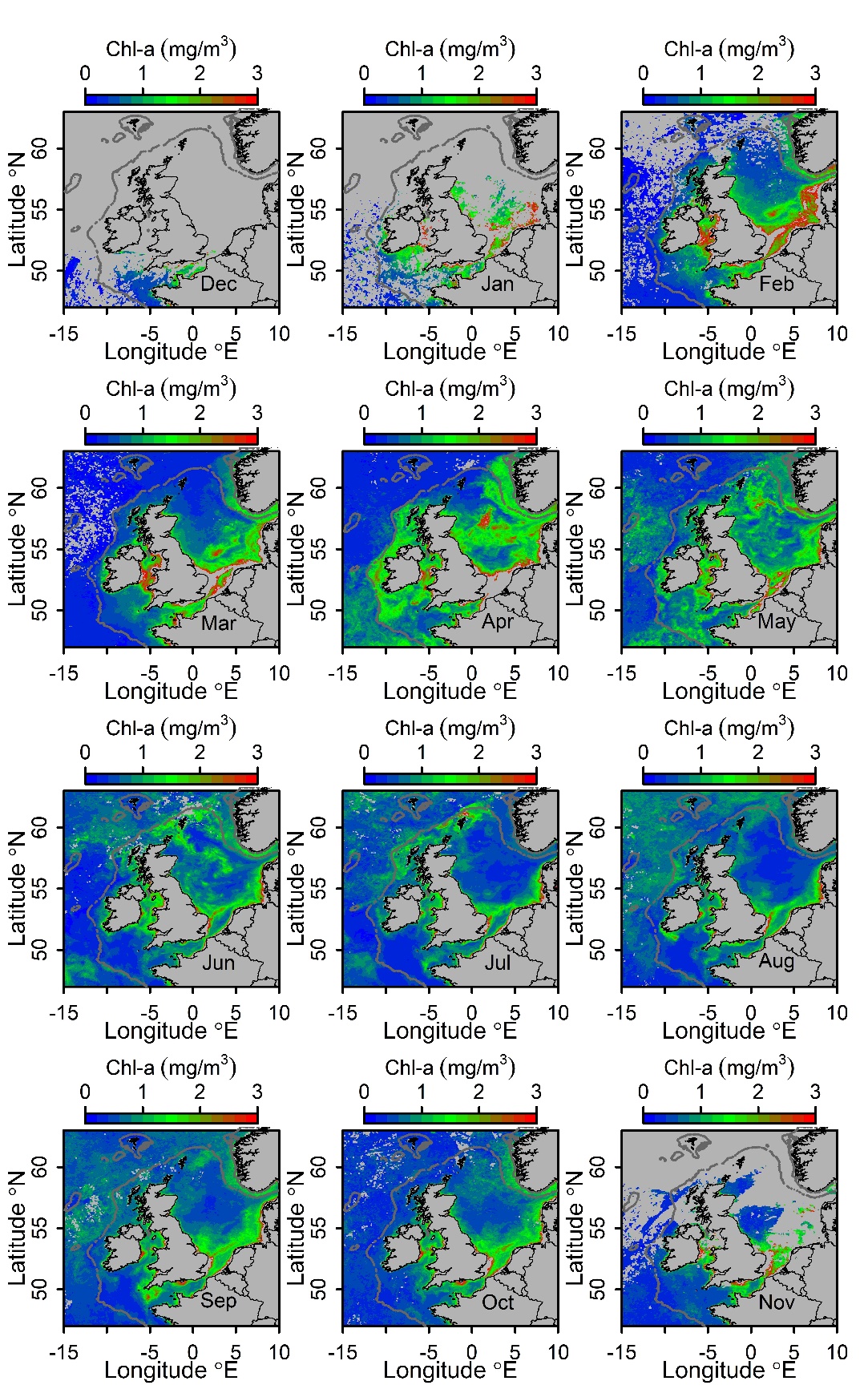


**Supplementary Figure 1:** Monthly mean Chlorophyll-a for the NW European shelf in 2015.


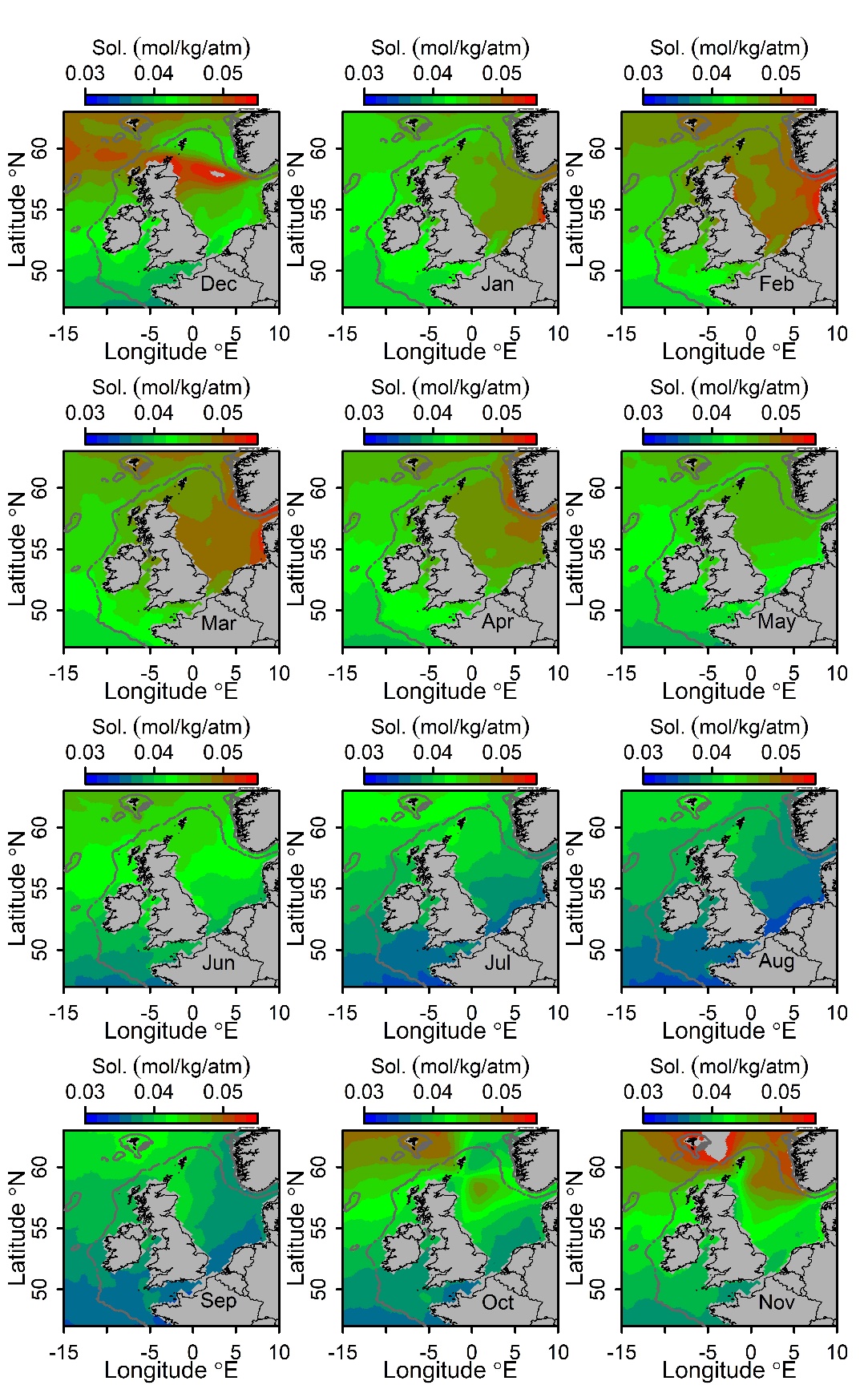


**Supplementary Figure 2:** Monthly mean CO_2_ solubility (α_sea_ from FluxEngine) for the NW European shelf in 2015.


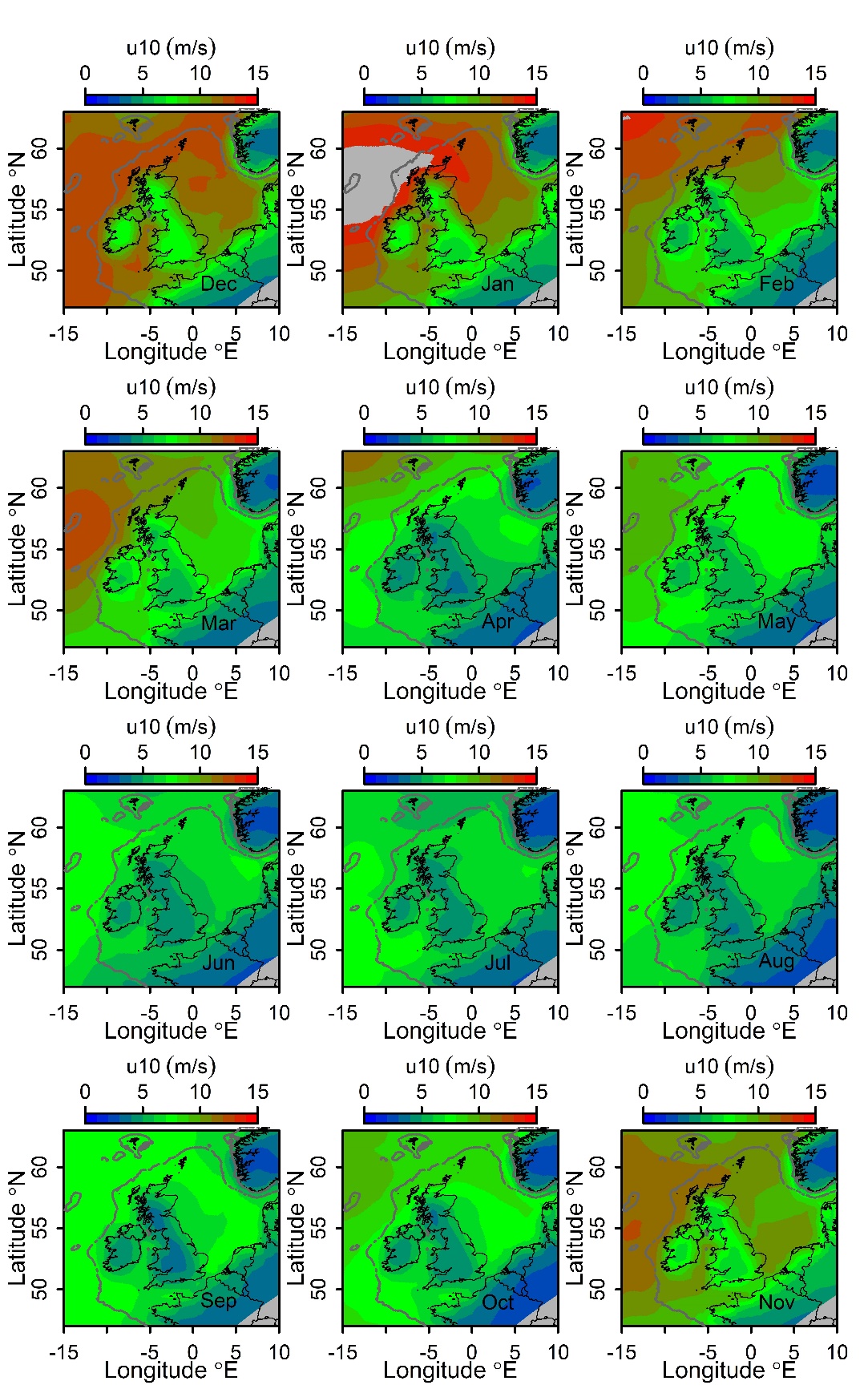


**Supplementary Figure 3:** Monthly mean wind speed at 10 m above sea-level (U10) for the NW European shelf in 2015.

**fCO_2_ method consistency – crossover analysis**

Each method for the determination of fCO_2_ carries a certain analytical uncertainty defined by its respective precision and accuracy, which, in turn, are influenced by properties such as instrument-drift, calibration frequency, equilibration timescale and resolution ^16,19^. In order to quantify the internal consistency of observations we examined ‘crossovers’ between different datasets and for the three method classes. A crossover was defined as a maximum distance of 40 km between observations, using 30 km as the equivalent distance for one day (e.g. two data-points, one day apart in time and 10 km apart in space, yield a nominal equivalent ‘distance’ between data-points of 40 km). This follows, but is stricter than, the SOCAT quality control criteria where a crossover is defined as a maximum distance of 80 km. We further refined the resulting crossovers by filtering the accepted crossovers to a maximum salinity and temperature difference of <0.2 and <0.5 ^o^C respectively. The crossover analysis was performed in three stages in R v3.4.1 ^20^: a) crossovers between individual equ-IR datasets and all other equ-IR datasets, b) crossovers between TA/DIC-derived and all equ-IR datasets and c) crossovers between sensor and all equ-IR datasets.

The crossover analysis revealed a high degree of consistency between the three different method classes (Supplementary Table 2). In all three cases, the linear slope (bias) for crossovers was statistically indistinguishable from unity. There was therefore no systematic bias by any of the measurement techniques, which allowed us to use the whole dataset for calculating air-sea fluxes. The crossover analysis was broadly consistent with numerous at-sea and laboratory inter-comparison exercises ^3,21-25^. However, all three method classes had a residual uncertainty which was 2- to 3-fold higher than their respective optimal method accuracy (Supplementary Table 2). This is likely because our analysis was not a controlled inter-comparison exercise where methods are tested in the same laboratory or ship using the same water. Given the relative contributions of each method class to the whole dataset and their respective uncertainties, we calculated a weighted uncertainty of 13.2 μatm for the whole dataset. For reference, the respective weighted accuracy of our dataset was 4 μatm.

Supplementary Table 2: Results of the method consistency analysis for fCO_2_ observations. The number of crossovers (*n*), correlation coefficient (R^2^), slope of the regression ± s.d. and mean absolute residual are given for a linear fit, applied to a) equ-IR pairs, b) TA/DIC-derived and equ-IR pairs and c) sensor and equ-IR pairs [equ-IR was the independent variable in all cases]. The individual method-class accuracy is listed for reference.

| Method-class | Crossovers (*n*) | R^2^ | Linear slope | Mean Abs. Res. (μatm) | Accuracy  (μatm) |
| --- | --- | --- | --- | --- | --- |
| equ-IR | 427 | 0.999 | 0.999±0.016 | 6.2 | 4 |
| TA/DIC-derived | 44 | 0.998 | 0.979±0.048 | 17.4 | 10 |
| sensor | 91 | 0.998 | 1.021±0.068 | 16.7 | 4 |
| all - weighted | - | - | - | 13.2 | 4 |

**Mapping uncertainty calculation**

In order to investigate uncertainties arising from the DIVA interpolation of fCO_2 sea_, we compared the interpolated outputs with independent fCO_2 sea_ data from a buoy in the Western English Channel (fCO_2 buoy_ at station L4 operated by PML and not included in the flux calculation). Since there were no *in situ* fCO_2 sea_ data for the L4 station in the collated dataset (for July-December 2015), fCO_2 buoy_ data were used to assess the interpolated DIVA output. The fCO_2 buoy_ data were derived from hourly pH-data (Satlantic, SeaFET) and TA as a function of salinity using the TA-salinity relationship for the L4 station ^7^. The computation of fCO_2 buoy_ was carried out using the CO2SYS software package as described above. The uncertainty of fCO_2 buoy_ (95 % c.i.) was calculated by propagating TA and pH uncertainty: two standard deviations of 9 μmol kg^-1^ and 0.007 pH units respectively. The 6-month continuous nature of this dataset practically eliminated temporal undersampling uncertainty. fCO_2 buoy_ data were binned into daily, then monthly bins and compared to the DIVA interpolated fCO_2_ data for the nearest grid cell (Supplementary Figure 1). The corresponding daily air to sea flux (F_buoy_) was computed using the same parameters as in FluxEngine (i.e. with the same *k*, α_sea_, α_air_, and fCO_2 air_), apart from the fCO_2 sea_ field. The daily flux was not significantly different from the FluxEngine output for the corresponding month (paired t-test; t=-9.34, *p*<0.001, *n*=184). Nevertheless, the 95 % confidence interval 0.003 mol m^-2^ month^-1^ represented an uncertainty of 16 % of the annual flux at L4 (0.201 mol m^-2^ y^-1^) when extrapolated for the whole year [i.e. 12 × 0.003 / 0.201 = 16 %]. We therefore attribute a mapping uncertainty of 16 % to our air to sea flux value.


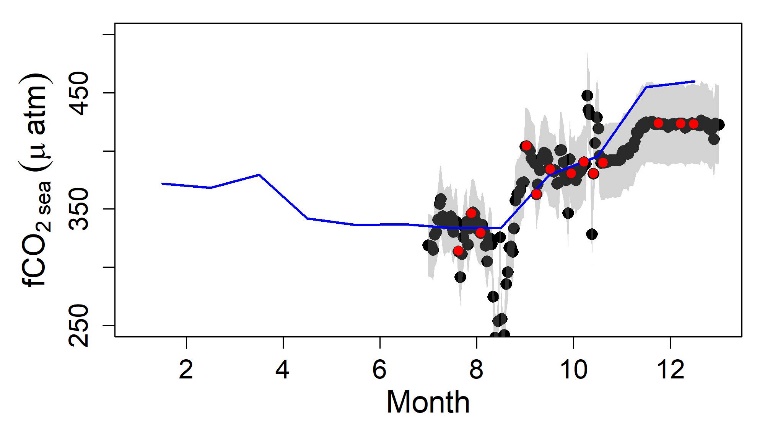


**Supplementary Figure 4:** Monthly fCO_2 sea_ (solid blue line) from the DIVA-interpolated output for the L4 station (50.25 ^o^N, 4.22 ^o^W). Additional data (not included in the collated fCO_2 sea_ dataset) are shown for fCO_2 sea_ calculated from discrete TA/DIC (red dots) and pH/TA from a buoy-mounted pH sensor (black dots). Shaded area represents the uncertainty (95 % c.i.) of the pH/TA-derived data.

**C-burial calculations**

C-burial in shelf sediments was calculated for three sites in the Celtic Sea: a) a muddy sediment (51.2114 ^o^N, 6.1338 ^o^W), b) a sandy sediment (51.0745 ^o^N, 6.5837 ^o^W) and c) a mud-sand sediment (station CCS: 49.4117 ^o^N, 8.5985 ^o^W). The following biogeochemical rates were determined at these sites in March (end of winter), May (during the spring bloom) and August 2015 (late summer): benthic oxygen consumption (Resp = respiration), nitrification (Nit), denitrification (Den), anammox (Ax) and sediment-water inorganic-N fluxes (FN; for NO_3_^-^+NO_2_^-^+NH_4_^+^) (e.g. Supplementary Table 3) ^26^. Our general reasoning for the C-burial calculation relies on the conservation of mass and specified C:N ratios in organic matter, where near-closure of the C-cycle is assumed *a priori* and tested against closure of the N-cycle *a posteriori* (see Supplementary Information for calculations).

Firstly, Resp, Nit, Den and Ax rates were transformed into units of C (Resp_C_, Nit_C_, Den_C_ and Ax_C_) using their respective respiratory quotients: R_q_=1 mol organic C produced per mol O_2_ consumed for Resp ^27-29^; R_q nit_=0.118 mol DIC consumed per mol N nitrified for Nit ^30,31^; R_q den_=1.436 mol organic-C remineralized per mol N denitrified for Den ^32-34^; R_q ax_=1.860 mol DIC consumed per mol N anammox for Ax ^33,35^.

Secondly, the sum of these C fluxes gave the organic-C consumed in sediments: Σ_orgC-consumed_ = Resp_C_ + Den_C_ – Nit_C_ – Ax_C_ [Nit and Ax are autotrophic (i.e. they consume DIC), hence the negative sign, while Resp and Den are heterotrophic processes (i.e. they consume organic C)]. Benthic oxygen consumption (Resp_C_) accounted for all of the Σ_orgC-consumed_ in our calculations (98 to 105 %).

Thirdly, various studies have shown a recycling efficiency in the order of 95-99 % in NW European shelf sea sediments ^36-38^. If we assume a recycling efficiency of 97 %, then Σ_orgC-consumed_ ~0.97 of organic-C deposited on sediments (C_dep_). The recycling efficiency of the sediments can then be independently verified by examining the N-cycle (if 3 % of C_dep_ is buried, then a corresponding amount of N must be buried with it). C_dep_ was converted to organic-N deposited on sediments (N_dep_) using a C:N ratio of 9.6 based on observations in the Celtic Sea and nearby English Channel ^7,37^.

Fourth, N_dep_ was compared to the return N-flux (sediments to water column; N_ret_=FN+Den+Ax). If N_dep_ exceeded N_ret_, then the difference was retained in sediments, i.e. buried N. This was converted to C-burial using a C:N ratio of 9.6 as above.

Using the sediment classification criteria of Folk ^39^ and data on the distribution of different sediments on the NW European shelf ^40^, we scaled these values over their respective sediment-types and areas. The uncertainty for this estimate was calculated from the sum of: a) propagating the standard error for the initial Resp, Nit, Den and Ax rates, b) increasing R_q_, R_q nit_, R_q den_ and R_q ax_ by 20% and c) reducing the C:N ratio to 6.7. These terms (a-c) accounted for 36 %, 30 % and 34 % of the total uncertainty respectively.

**Supplementary Table 3:** Data used for the C-burial calculation terms for the mud-sand site in the central Celtic Sea (station CCS: 49.4117 ^o^N, 8.5985 ^o^W). Benthic oxygen consumption (Resp) in mmol O_2_ m^-2^ d^-1^, nitrification (Nit), denitrification (Den), anammox (Ax) and sediment-water nutrient fluxes (FN) in mmol N m^-2^ d^-1^. Organic-C deposition (C_dep_; in mmol C m^-2^ d^-1^), organic-N deposition (N_dep_; in mmol N m^-2^ d^-1^) calculated from C_dep_ with a C:N ratio of 9.6. The return flux of DIN+N-losses due to denitrification and anammox (N_ret_; in mmol N m^-2^ d^-1^) and N_ret_ as a percentage of N_dep_.

| Date | Resp | Den | Nit | Ax | FN | C_dep_ | N_dep_ | N_ret_ | % N_ret_/N_dep_ |
| --- | --- | --- | --- | --- | --- | --- | --- | --- | --- |
| Mar | 1.5 | 0.03 | 0.09 | 0.01 | 0.09 | 1.52 | 0.16 | 0.14 | 84 |
| May | 2.8 | 0.08 | 1.78 | 0.02 | 0.19 | 2.66 | 0.28 | 0.34 | 117 |
| Aug | 4.5 | 0.12 | 1.89 | 0.03 | 0.17 | 4.37 | 0.46 | 0.38 | 80 |

**Supplementary References**

1 Hartman, S. E. *et al.* Seasonality and spatial heterogeneity of the surface water carbonate system on the NW European shelf. *Progress in Oceanography*, doi: 10.1016/j.pocean.2018.1002.1005, doi:10.1016/j.pocean.2018.02.005 (2019).

2 Jones, E. M. *et al.* Mesoscale features create hotspots of carbon uptake in the Antarctic Circumpolar Current. *Deep Sea Research Part II: Topical Studies in Oceanography* **138**, 39-51, doi:<https://doi.org/10.1016/j.dsr2.2015.10.006> (2017).

3 Ribas-Ribas, M. *et al.* Intercomparison of carbonate chemistry measurements on a cruise in northwestern European shelf seas. *Biogeosciences* **11**, 4339-4355, doi:10.5194/bg-11-4339-2014 (2014).

4 Pierrot, D. *et al.* Recommendations for autonomous underway pCO(2) measuring systems and data-reduction routines. *Deep-Sea Res. Part II-Top. Stud. Oceanogr.* **56**, 512-522, doi:10.1016/j.dsr2.2008.12.005 (2009).

5 Petersen, W. FerryBox systems: State-of-the-art in Europe and future development. *J. Mar. Syst.* **140**, 4-12, doi:10.1016/j.jmarsys.2014.07.003 (2014).

6 Lefevre, N., Diverres, D. & Gallois, F. Origin of CO2 undersaturation in the western tropical Atlantic. *Tellus Series B-Chemical and Physical Meteorology* **62**, 595-607, doi:10.1111/j.1600-0889.2010.00475.x (2010).

7 Kitidis, V. *et al.* Seasonal dynamics of the carbonate system in the Western English Channel. *Continental Shelf Research* **42**, 30-40, doi:

doi: /10.1016/j.csr.2012.04.012 (2012).

8 Marrec, P. *et al.* Seasonal and latitudinal variability of the CO2 system in the western English Channel based on Voluntary Observing Ship (VOS) measurements. *Marine Chemistry* **155**, 29-41, doi:10.1016/j.marchem.2013.05.014 (2013).

9 Olsen, A., Brown, K. R., Chierici, M., Johannessen, T. & Neill, C. Sea-surface CO(2) fugacity in the subpolar North Atlantic. *Biogeosciences* **5**, 535-547, doi:10.5194/bg-5-535-2008 (2008).

10 Schuster, U. & Watson, A. J. A variable and decreasing sink for atmospheric CO2 in the North Atlantic. *J. Geophys. Res.-Oceans* **112**, doi: 10.1029/2006jc003941, doi:C11006

10.1029/2006jc003941 (2007).

11 Frankignoulle, M., Borges, A. & Biondo, R. A new design of equilibrator to monitor carbon dioxide in highly dynamic and turbid environments. *Water Research* **35**, 1344-1347, doi:10.1016/s0043-1354(00)00369-9 (2001).

12 Lewis, E. & Wallace, D. W. R. Program Developed for CO_2_ System Calculations., ORNL/CDIAC-105. (Carbon Dioxide Information Analysis Center, Oak Ridge National Laboratory, U.S. Department of Energy, Oak Ridge, Tennessee., 1998).

13 Dickson, A. G. Standard potential of the reaction -AgCl(s)+1/2H-2(g)=Ag(s)+HCl(aq) and the standard acidity constant of the ion HSO4- in synthetic sea-water from 273.15-K to 318.15-K. *Journal of Chemical Thermodynamics* **22**, 113-127, doi:10.1016/0021-9614(90)90074-z (1990).

14 Dickson, A. G. & Millero, F. J. A Comparison of the Equilibrium-Constants for the Dissociation of Carbonic-Acid in Seawater Media. *Deep-Sea Research Part a-Oceanographic Research Papers* **34**, 1733-1743 (1987).

15 Mehrbach, C., Culberso.Ch, Hawley, J. E. & Pytkowic.Rm. Measurement of Apparent Dissociation-Constants of Carbonic-Acid in Seawater at Atmospheric-Pressure. *Limnology and Oceanography* **18**, 897-907 (1973).

16 Dickson, A. G., Sabine, C. L. & Christion, J. R. Guide to best practices for ocean CO_2_ measurements. PICES Special Publication 3. 191 (2007).

17 Orr, J. C., Epitalon, J.-M., Dickson, A. G. & Gattuso, J.-P. Routine uncertainty propagation for the marine carbon dioxide system. *Marine Chemistry* **207**, 84-107, doi:<https://doi.org/10.1016/j.marchem.2018.10.006> (2018).

18 Fietzek, P., Fiedler, B., Steinhoff, T. & Kortzinger, A. In situ Quality Assessment of a Novel Underwater pCO(2) Sensor Based on Membrane Equilibration and NDIR Spectrometry. *Journal of Atmospheric and Oceanic Technology* **31**, 181-196, doi:10.1175/jtech-d-13-00083.1 (2014).

19 Wanninkhof, R. *et al.* Incorporation of alternative sensors in the SOCAT database and adjustments to dataset Quality Control flags. (Carbon Dioxide Information Analysis Center, Oak Ridge National Laboratory, US Department of Energy Oak Ridge, Tennessee, <http://cdiac.ornl.gov/oceans/Recommendationnewsensors.pdf>, doi:10.3334/CDIAC/OTG.SOCAT_ADQCF, 2013).

20 R-Core-Team. *R: A language and environment for statistical computing.*, (R Foundation for Statistical Computing, 2014).

21 Körtzinger, A. *et al.* The international at-sea intercomparison of fCO_2_ systems during the R/V Meteor Cruise 36/1 in the North Atlantic Ocean. *Marine Chemistry* **72**, 171-192, doi:10.1016/s0304-4203(00)00080-3 (2000).

22 Körtzinger, A. *et al.* At-sea intercomparison of two newly designed underway pCO(2) systems - Encouraging results. *Marine Chemistry* **52**, 133-145, doi:10.1016/0304-4203(95)00083-6 (1996).

23 Lamb, M. F. *et al.* Consistency and synthesis of Pacific Ocean CO2 survey data. *Deep-Sea Res. Part II-Top. Stud. Oceanogr.* **49**, 21-58, doi:10.1016/s0967-0645(01)00093-5 (2002).

24 Lueker, T. J., Dickson, A. G. & Keeling, C. D. Ocean pCO(2) calculated from dissolved inorganic carbon, alkalinity, and equations for K-1 and K-2: validation based on laboratory measurements of CO2 in gas and seawater at equilibrium. *Marine Chemistry* **70**, 105-119, doi:10.1016/s0304-4203(00)00022-0 (2000).

25 Millero, F. J. *et al.* Dissociation constants for carbonic acid determined from field measurements. *Deep-Sea Res. Part I-Oceanogr. Res. Pap.* **49**, 1705-1723 (2002).

26 Kitidis, V. *et al.* Seasonal benthic nitrogen cycling in a temperate shelf sea: the Celtic Sea. *Biogeochemistry* **135**, 103-119, doi:10.1007/s10533-017-0311-3 (2017).

27 Forja, J. M., Ortega, T., DelValls, T. A. & Gomez-Parra, A. Benthic fluxes of inorganic carbon in shallow coastal ecosystems of the Iberian Peninsula. *Marine Chemistry* **85**, 141-156, doi:10.1016/j.marchem.2003.09.007 (2004).

28 Glud, R. N. Oxygen dynamics of marine sediments. *Mar. Biol. Res.* **4**, 243-289, doi:10.1080/17451000801888726 (2008).

29 Thouzeau, G. *et al.* Spatial and temporal variability of benthic biogeochemical fluxes associated with macrophytic and macrofaunal distributions in the Thau lagoon (France). *Estuar. Coast. Shelf Sci.* **72**, 432-446, doi:10.1016/j.ecss.2006.11.028 (2007).

30 Ebeling, J. M., Timmons, M. B. & Bisogni, J. J. Engineering analysis of the stoichiometry of photoautotrophic, autotrophic, and heterotrophic removal of ammonia-nitrogen in aquaculture systems. *Aquaculture* **257**, 346-358, doi:10.1016/j.aquaculture.2006.03.019 (2006).

31 Ge, S. J. *et al.* Detection of nitrifiers and evaluation of partial nitrification for wastewater treatment: A review. *Chemosphere* **140**, 85-98, doi:10.1016/j.chemosphere.2015.02.004 (2015).

32 Gomez, M. A., Gonzalez-Lopez, J. & Hontoria-Garcia, E. Influence of carbon source on nitrate removal of contaminated groundwater in a denitrifying submerged filter. *J. Hazard. Mater.* **80**, 69-80, doi:10.1016/s0304-3894(00)00282-x (2000).

33 Kumar, M. & Lin, J. G. Co-existence of anammox and denitrification for simultaneous nitrogen and carbon removal-Strategies and issues. *J. Hazard. Mater.* **178**, 1-9, doi:10.1016/j.jhazmat.2010.01.077 (2010).

34 Reyes-Avila, J. S., Razo-Flores, E. & Gomez, J. Simultaneous biological removal of nitrogen, carbon and sulfur by denitrification. *Water Research* **38**, 3313-3321, doi:10.1016/j.watres.2004.04.035 (2004).

35 Tomaszewski, M., Cema, G. & Ziembinska-Buczynska, A. Influence of temperature and pH on the anammox process: A review and meta-analysis. *Chemosphere* **182**, 203-214, doi:10.1016/j.chemosphere.2017.05.003 (2017).

36 de Haas, H., van Weering, T. C. E. & de Stieger, H. Organic carbon in shelf seas: sinks or sources, processes and products. *Continental Shelf Research* **22**, 691-717, doi:10.1016/s0278-4343(01)00093-0 (2002).

37 Humphreys, M. P. *et al.* Mechanisms for a nutrient-conserving carbon pump in a seasonally stratified, temperate continental shelf sea. *Progress in Oceanography*, doi: 10.1016/j.pocean.2018.1005.1001, doi:10.1016/j.pocean.2018.05.001 (2019).

38 Middelburg, J. J. Reviews and syntheses: to the bottom of carbon processing at the seafloor *Biogeosciences* **15**, 413-427, doi:10.5194/bg-15-413-2018 (2018).

39 Folk, R. L. The Distinction between Grain Size and Mineral Composition in Sedimentary-Rock Nomenclature *The Journal of Geology* **62**, 344-359 (1954).

40 Wilson, R. J., Speirs, D. C., Sabatino, A. & Heath, M. R. A synthetic map of the north-west European Shelf sedimentary environment for applications in marine science. *Earth System Science Data* **10**, 109-130, doi:10.5194/essd-10-109-2018 (2018).
